# Shining the Light on Senescence Associated LncRNAs

**DOI:** 10.14336/AD.2016.0810

**Published:** 2017-04-01

**Authors:** A.R. Ghanam, Qianlan Xu, Shengwei Ke, Muhammad Azhar, Qingyu Cheng, Xiaoyuan Song

**Affiliations:** ^1^CAS Key Laboratory of Brain Function and Disease, CAS Center for Excellence in Molecular Cell Science, Collaborative Innovation Center of Chemistry for Life Sciences, School of Life Sciences, University of Science and Technology of China, Hefei 230027, China.; ^2^Collage of Veterinary Medicine, Suez Canal University, Ismailia 41522, Egypt

**Keywords:** cellular senescence, lncRNAs, biomarkers

## Abstract

Cellular senescence can be described as a complex stress response that leads to irreversible cell cycle arrest. This process was originally described as an event that primary cells go through after many passages of cells during cell culture. More recently, cellular senescence is viewed as a programmed process by which the cell displays a senescence phenotype when exposed to a variety of stresses. Cellular senescence has been implicated in tumor suppression and aging such that senescence may contribute to both tumor progression and normal tissue repair. Here, we review different forms of cellular senescence, as well as current biomarkers used to identify senescent cells *in vitro* and *in vivo*. Additionally, we highlight the role of senescence-associated long noncoding RNAs (lncRNAs).

## Senescence: past and present

Cellular senescence was first discovered *in vitro* as a state of irreversible cell cycle arrest [1]. In the ensuing decades, researchers have described different causes for cellular senescence as well as the nature of the senescent phenotype. Currently, they are using different methods to elucidate the mechanisms that underlie the different forms of senescence that have been observed, starting with replicative senescence and telomere association. Others have studied mechanisms of senescence by using different methods to induce senescence, usually by exposing the cells to different stressors. Importantly, we are beginning to understand the molecular relevance of senescence. Until now, the prevailing view was that senescence occurs only under pathological conditions. However, recently, Storer et al. described a role for cellular senescence during development [2, 3]. They observed the widespread appearance of senescent cells in distinct patterns and specific stages of developing vertebrate embryos in chick [3] and human embryos [2], suggesting that developmental senescence is a conserved feature of vertebrate embryonic development.

**Figure 1.**
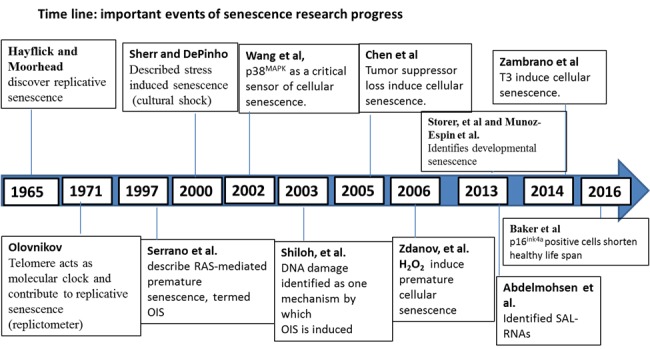
Timeline of pivotal steps in the study of senescence.

As it turns out, the observed developmentally-programmed senescence pathway is mediated by p21. This pathway appears to be independent of DNA damage or other mediators associated with senescence, including p53. One particularly interesting aspect that both studies pointed out is the connection between senescence and apoptosis, but the underlying mechanism remains obscure. The following time line describes some of the pivotal points in the study of senescence (Fig. 1).

Whatever the cause of senescence, cells not only exhibit a marked alteration in their transcriptome and the secretion of many factors including cytokines and chemokines, but also acquire a characteristic phenotype [4].

## General features of senescent cells

Non-dividing (arrested) cells can either be reversible quiescent or irreversible senescent, although these features are not always distinctive. Senescent cells stop division like quiescent cells, but no longer possess the proliferative potential. The following points cover the main features of senescent cells.

### Growth arrest

The senescent state is essentially permanent and cannot be reversed by known cell-division stimuli. CDK p16^INK4a^ enforces the cell-cycle arrest through activation of the tumor suppressor Rb. Some senescent cells that do not express CDK p16^INK4a^ can resume growth after genetic interventions to inactivate the tumor suppressor p53 [5]. The role of P53 in senescence and longevity is complex; it was believed to be a senescence inducer, and its ablation has allowed the cell to return to cell cycle progression [6-8]; however, recent studies indicate that P53 may act as anti-aging factor in some conditions when mTOR is inhibited by suppressing cellular senescence, converting it into quiescence [9]. In conclusion, senescent cells have permanent loss of proliferative potential [10, 11].

### Large and flat morphology

Senescent cells increase in size (hypertrophy), sometimes enlarge more than two-fold relative to the size of non-senescent counterparts. They also have a flat, vacuolated morphology and are unable to synthesize DNA [1, 12].

### Contribute to aging and tissue healthiness

Senescent cells acquire the characteristics that may compromise normal tissue functions, and their accumulation in the later life might lead to organismal aging and engage to the appearance of age-related pathologies as atherosclerosis [4, 13]. The accumulation of p16^Ink4a^ positive senescent cells induces tissue degeneration and cataracts in mice, while the clearance of p16^Ink4a^ senescent cells delays aging and increases the overall health of mice. These findings suggested that the accumulation of p16^Ink4a^-expressing cells during normal aging also decreases the overall health of the cells [14, 15].

**Figure 2.**
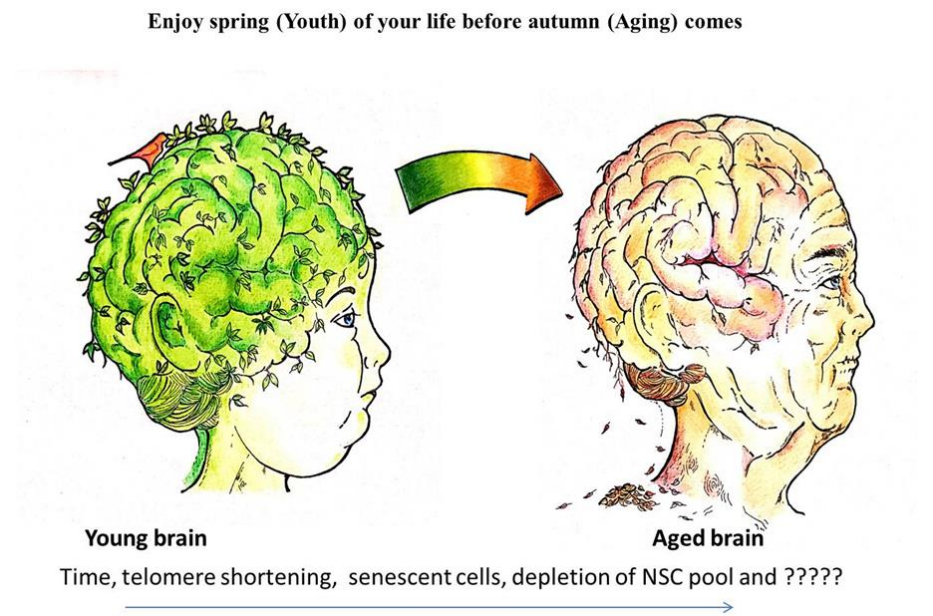
A photograph of the brain aging with time, telomere shortening, senescent cells or depletion of adult neural stem cells. Do we think we are able to keep our brain green in spite of time passing? Are we only able to delay the onset of our brain autumn and if we succeed in delaying it, will our life span be increased or only cellular function (healthiness) be improved during our limited lifespan?

Senescent cells might drive aging via one of three possible mechanisms. First, the rise in the number of senescent cells may be due to increased accumulation, decreased clearance, depletion of the available pool of stem cells, or a combination of some or all of these mechanisms (Fig. 2). These factors will compromise tissue repair, regeneration, and normal turnover, leading to functional impairment [14]. Second, senescent cells secrete factors that affect vital processes such as cell growth and migration, tissue architecture, blood vessel formation, and differentiation. These factors are highly regulated. The presence of excessive amounts of these factors can cause disruption of tissue structure and function. Third, the senescent associated secretory phenotype (SASP) includes several potent inflammatory cytokines [15]. Low-level, chronic, “sterile” inflammation is a hallmark of aging that initiates or promotes most, if not all, major age-related disease [16, 17]. Inflammation produced by SASP has been observed in patients with neurodegeneration, who present high expression of interleukin-1 and interleukin-6 [18-20] and with atherosclerosis [21], type 2 diabetes mellitus and obesity [22].

Because some immune cells produce strong oxidants, chronic inflammation can result in cell and tissue destruction. Also, immune cells can further alter the tissue environment through secretion of additional factors that can induce cell and tissue dysfunction and damage stem cell populations. Inflammatory, oxidative damage can also initiate carcinogenesis, and the inflammatory milieu can promote cancer by suppressing immune surveillance and stimulating malignant phenotypes [23, 24].

### DNA damage and DDR

Maintenance of the genome integrity is critical for the development of the organism and prevention of genetic disorders including some forms of cancer. Eukaryotic cells have developed a specialized DNA damage response (DDR) to protect against alterations of the genome as a result of both extrinsic and intrinsic genotoxic insults.

Cellular senescence is a state of irreversible cell cycle arrest, typically driven by a persistent DDR [25]. Genotoxic insults either in telomeric or non-telomeric regions, considered as DNA DSBs, also contribute to senescence [26]. Senescent cells with persistent DSBs activate DDR machinery network of proteins kinases such as ATM, ATR and DNA-PKCs, which in turn, attach to DNA segments and form persistent foci (DNA-SCRAS) [27] [11, 28], and are distinguishable from transient damage foci [29]. DNA-SCARS include dysfunctional telomeres that have been identified as DNA DSBs [27, 28, 30, 31].

### Transcriptome changes

Senescent cells exhibit profound changes in their transcriptomes. A major consequence of these changes is the secretion of many factors, including cytokines and chemokines, growth factors, proteases [4, 32-35], and SA-β-gal [12], which partly reflects the increase in lysosomal mass [36] as well as p16^INK4a^, which also is not commonly expressed by quiescent or terminally differentiated cells [37-41]. Also, senescent cells activate downstream signaling pathways that trigger the production and release of reactive oxygen species (ROS) as well as a wide variety of other bioactive molecules [42].

Senescent cells express surface-bound ligands and adhesion molecules, which enable natural killer and other immune cells to attack them [24], although it is not known when these proteins are expressed relative to the SASP. Another unclear issue is whether the generation rate is higher than that of clearance in aged individuals or do both contribute to aging. Recently, a major difference was also found in RNA-binding proteins as well as ncRNAs between senescent and non-senescent cells.

All these transcriptome changes affect the extracellular matrix and have potent autocrine and paracrine influence on the activity of neighboring cells, and it is viewed as the nidus for all aging-associated diseases and senescence phenotype.

## General roles of lncRNAs in senescence

LncRNAs are noncoding transcripts ≥ 200 nt, lack ORF, and may originate from mutations in a protein coding genes, chromosomal rearrangements, or duplications in a ncRNA sequence and transposable element insertion. The exact roles of these heterogeneous transcripts rely mainly on their associated partner molecules, which may be RNA or DNA or protein. Although many lncRNAs have been identified, not more than handful of lncRNAs have been characterized functionally, and many of them wait for further verification.

Before surveying their molecular mechanism in senescence, it is helpful to summarize the general functions of lncRNAs discovered in senescence. They regulate gene expression via diverse mechanisms at both the transcriptional level and the post-transcriptional level, and the regulatory models of lncRNAs were discussed in details in the review of [43].

### Regulation of chromatin structure

Regulation of chromatin structure that causes gene silencing by lncRNAs can occur in two ways. The first is in the cis formation by coating gene clusters, which renders them inaccessible to transcription by the actions of lncRNAs such as *Xist* and *Air*. The other is in the trans formation whereby lncRNAs, such as *HOTAIR*, interact with chromatin modifying proteins and epigenetically silence genes at another locus. Interestingly, *HOTAIR* was found to be highly up-regulated in senescent cells [44].

### Transcription of lncRNAs themselves

This involves activating transcription of other protein coding genes by opening the chromatin structure to allow access of transcription machinery into a specific genetic locus. For example, transcription of lncRNAs *UAS1* and *UAS2* has been shown to activate the expression of the *FBP1* gene. Transcription of lncRNAs near protein-coding loci can also repress gene transcription. In this case, lncRNA *SRG1* has been shown to inhibit transcription of the overlapping *SER3* gene [45, 46].

### Transcription factor regulation

Through allosteric interactions, lncRNA transcripts can activate various transcription factors, including activation of the *Dlx5/6* enhancer, through transcription factor *DLX2*, by the lncRNA *Evf2*, which plays an important role in the forebrain development and neurogenesis of interneurons that produce GABA [47]. LncRNAs can bind to accessory proteins to activate them allosterically, or induce their oligomerization leading to the activation, as in the case of lncRNA-induced trimerization of HSF1 proteins in response to heat shock [45, 46]. Further, critical movement of transcription factors within the cell can be altered by lncRNA transcripts, which can enhance access to binding sites or prevent access, as has been observed with lncRNA NRON, which prevents the transcription factor NFAT from entering the nucleus [45, 46].

### Post-transcriptional modifications

Some lncRNAs are actually antisense to certain protein-coding genes. These lncRNAs may regulate the splicing, editing, transport, translation, or degradation of the corresponding mRNA transcripts.

### Small ncRNAs precursors

LncRNAs can be precursors of small ncRNAs such as siRNAs or miRNAs, which function to down-regulate gene expression through degradation of mRNA transcripts or repression of translation. LncRNA *H19* is one example. In ESC and placenta, *H19* is processed within the cell to become the microRNA, miR-675 which is important in embryonic development, and it is also implicated in growth, proliferation, cell cycle, apoptosis, and aging [48, 49]. The levels of *H19* were further elevated in old prostate tissues and may be associated with various diseases including cancers [50, 51]. For detailed lncRNAs regulation at different levels of gene expression including transcriptionally and post-transcriptionally please check the review of [52].

## Functions of lncRNAs in different types of senescence

### Cellular senescence

Telomeres are structures that protect the ends of chromosomes against damage and shortening of telomeres is one of the major mechanisms leading to cellular senescence (the so-called end replication problem) [53]. Further, telomeres act as a molecular clock, reflecting the replicative history of a primary cell and telomere length is regulated by telomerase ribonucleoprotein complex (shelterin protein complex and the lncRNA *TERC*). The lncRNA *TERC* is directly implicated in maintenance of telomere length and thus delays the onset of aging. *TERC* deficient mice showed short telomeres, chromosomal instability, and premature aging [54].

Some lncRNAs were altered in replicative senescence. Abdelmohsen et al. identified a number of characterized and uncharacterized lncRNAs that were dysregulated in senescent cells, among which *MALAT1* showed down-regulation [55]. Furthermore, *XIST*, a lncRNAs that is responsible for X chromosome inactivation in females, was shown to be declined in senescent cells [51, 56], but its specific function is still unknown. Also, our group identified a number of dysregulated lncRNAs in replicative senescence of MEF cells (unpublished data).

### Induced senescence

A range of conditions *in vitro* can induce senescence. If telomere loss or dysfunction is not observed, this type of senescence is designated as premature, since it arises before the occurrence of telomere shortening. Evidence for the existence of *in vivo* premature senescence *in vivo* has been mounting. The involvement of some biomarkers such as p16/ARF, which has a critical role in tumor suppression, has been reported in some induced senescence. Senescence can be induced by one of the following: stress, oncogene, H_2_O_2_ and thyroxin (T3). In the following section, we will focus on oncogene-induced senescence where lncRNAs were addressed.

### Oncogene-induced senescence (OIS)

OIS is thought to protect tissues from tumor progression and associated with human benign and precancerous lesions. Overexpression of oncogenic Ras, a cytoplasmic mitogenic signal in normal human fibroblasts, produces a replicative senescence phenotype (cell cycle arrest), with positive senescence biomarkers such as SA-β-gal stain but without telomere shortening [37, 57-59], and with activation of DNA DSB checkpoint kinases: ATM , ATR and DNA-PKcs [37, 59-63] as well as increased expression of tumor suppressor p16^INKA4^ , ARF, p21^Waf1^ and p53 that induce cell cycle arrest [37, 60, 61, 64, 65]. The link between endogenous Ras in tumor lines and the level of p53 confirms its role in senescence [66, 67]. As DNA DSBs are one hallmark of senescence, the lncRNA-*JADE* transcriptionally activates Jade1 and induces histone H4 acetylation in the DDR [68].

*ANRIL* (Antisense Noncoding RNA in the INK4 Locus) is a lncRNA transcribed from CDKN2B locus in the antisense orientation. It was found to be down-regulated in replicative senescence and oncogenic RAS overexpression [69-71].

The relationship of tumor suppressors and senescence has been assessed in mouse and human cells *in vitro*. It was shown that MEFs fully deficient of PTEN underwent senescence, which was accompanied by induction of p53 without DDR. In human fibroblast senescence, the functional significance of p53 is somewhat eclipsed by p16^Ink4a^, which governs irreversible phenotype and conditional activation of p53 in p53 deficient human tumor cells was found to promote irreversible cell cycle arrest with senescence features [72, 73].

The lncRNA *7SL* regulates P53 mRNA translation and MEG3 can regulate P53 directly via RNA-protein association or indirectly by lowering MDM2 expression levels [74, 75].

Recent individual studies also indicate the potential roles of lncRNAs in senescence. RNA-Seq and microarray data have identified altered levels of lncRNAs during aging in response to various types of senescence stimuli (Table 1). Several studies indicate that some lncRNAs were induced in a P53-dependent manner [76, 77]. Furthermore, P53 may bind to enhancer regions, promoting the expression of a new class of lncRNA called enhancer RNAs (eRNAs). These eRNAs acts as transcriptional regulators of P53 target genes [78].

The overexpression of oncogenic RAS leads to changes of lncRNAs expression profile. Among these lncRNAs, *BANCER* (BRAF-activated lncRNA) regulates the migration invasion and proliferation of tumor [70, 79] and the lncRNA *VAD* was up-regulated on OIS and activates CDKN2A locus, and this promotes senescence. *VAD* decreases the occupancy of the repressive variant histone H2A.Z at the CDKN4A promoters thereby allowing the expression of p16 and p14 [80]. However, the direct effect of these lncRNAs in senescence is still unclear, and the exact mechanism is still missing.

As previously mentioned the key role of P16 in senescence, the stability of its mRNA was regulated by lncRNA *UCA1* by sequestering *hnRNAP1*, and its overexpression induces senescence, while its down regulation delays the onset of senescence [81]. Furthermore, *SALNR* was observed to be down-regulated in both replicative and OIS [82]. It interacts with NF90, a nuclear protein that prevents the biogenesis of senescence-associated miRNAs [83]. Overexpression of *SALNR* inhibits the localization of NF90 to the nucleoli, which delays the onset of senescence.

*FAL-1* (Focally amplified lncRNA on chromosome 1) was found to be increased in ovarian cancer as an oncogenic lncRNA and interacts with BMI1, PRC1 component, resulting in repression of p21. Consequently, knocking down of *FAL-1* promotes senescence via inducing p21 expression [84].

**Table 1:** LncRNAs affecting molecular traits in cell cycle of cellular senescence

LncRNA	Role is senescence	Cell cycle effect	Citation
MALAT 1	Inhibit senescence, promote cell division	G0	[85]
Gadd7	Binds TDP-43, lowers Cdk6 mRNA	G1/S	[86]
MEG3	Upregulates p53, blocks apoptosis	N/A	[87]
7SL	Inhibits senescence, suppresses p53	G1, G2, S	[88]
eRNAs	p53-regulated, induce cellular senescence	G1	[78]
UCA1	Inhibits senescence, inhibits senescence cdki p27	G1	[89]
SRA	Inhibits senescence cdki p21, p27	G2, M	[90]
ANRIL	Prevents expression of p15 and p16	N/A	[69]
Kcnq 1OT1	Cell division and DNA methylation	N/A	[48]
NcRNACCND1	Inhibits transcription of *CCND1* gene	G1,S	[91, 92]
HOTAIR	Scaffold for MEX3 and DZIP3 E3 ubiquitin Ligase	G2, G1	[44, 93]
TERRA	Enhance mobility of shelterin proteins to keep Telomere stability	N/A	[94]
AsncmtRNA2	Up regulated in replicative senescence	N/A	[95]

## Roles of RBPs in senescence

RBP are proteins that bind to RNA in cells and participate in forming of ribonucleoprotein complexes, cytoplasmic or nuclear located. RBPs are involved in a range of cellular processes and play a major role in the post-transcriptional control of RNAs, such as splicing, polyadenylation, mRNA stabilization, transportation, localization and translation.

RBPs, such as human antigen R (HuR), AU-binding factor 1 (AUF1), and tristetraprolin (TTP), can influence cellular senescence through association with mRNAs that encode various senescence factors [96-99]. The HuR promoted HOTAIR decay and loss of HuR during senescence may contribute to its stabilization and subsequent up-regulation [100]. Further, The RNA bound HuR can displace 7SL, which binds to TP53 mRNA and suppress P53 translation, thus enhance P53 translation. According to this competitive interaction, when 7SL was silenced, HuR binding to TP53 mRNA was increased, which subsequently promoted p53 translation, resulting in increased cell cycle arrest and senescence [88].

The lncRNA *Gadd7*, induced by DNA damage and oxidative stress in Chinese Hamster ovary, controls G1/S checkpoint and cell growth [101]. Further, Gadd7 is associated with RBP TDP-43 (TAR DNA-binding protein) and interferes with the binding of TDP-43 to Cdk6 mRNA, leading to destabilization of Cdk6 mRNA, abnormal cell cycle progression and incidence of senescence [86, 102]. The exact role of *Gadd7* in senescence is still unknown.

The lncRNA *GAS5* acts as a decoy for the transcription factor glucocorticoid receptor (GR) such that the presence of *GAS5* produces growth arrest through inhibition of GR-mediated gene expression [103]. Specifically, GAS5 prevents mobilization of GR protein from the cytosol to the nucleus [104].

p53-dependent DNA damage stimulates expression of the lncRNA *PANDA*, which binds with the transcription factor NF-YA and interferes with its transcriptional activity, lowering the expression of apoptotic genes. Interestingly, NF-YA’s interaction with P53 impacts upon cell cycle regulation and senescence [105, 106]. These findings indicated that PANDA could be involved in DNA damage-induced senescence through NF-YA and P53.

*NORAD*, another DNA damage activated lncRNAs, was found to maintain genomic stability by sequestering of Pumilio proteins during chromosomal segregation [107]. While pumilio-Fem3-binding factor (PUF) protein acts as a negative regulator of gene expression [108] by binding to 3’ UTR of target mRNA through their PUMILIO homology domain and enhances deadenylation and decapping, resulting in an accelerated turnover and decreased translation [109, 110].

LncRNAs have been linked to a wide range of cellular functions that require their interaction with one or more RBPs. RBPs also typically bind to many different RNAs. A clearer picture of the intricate network of interactions, whose deregulation is frequently associated with disease pathophysiology is currently emerging. Several new techniques have been developed recently to obtain protein-RNA binding data in a high-throughput fashion. It is postulated that very soon protein-lncRNA interaction networks will be described which are likely to offer important clues for increased understanding of the cellular functions of lncRNAs and their disease-associated perturbations.

## Short list of the common senescence biomarkers

Biomarkers for senescent cells must have the following merits: specific to senescent cell but not quiescent nor presenescent cells, increase in an age-dependent manner, expressed in benign tumors or premalignancies, may be related to different pathways of induced senescence, available for *in vitro* and *in vivo* detection, can be used to detect different forms of senescence and easy to perform in a simple way. Disappointedly, there is until now no ideal biomarker for all cases of senescence, and the combination of different “preliminary” markers must be followed by molecular confirmation. Here we list the most commonly used preliminary biomarkers for senescence.

### Senescence associated β-Galactosidase

The SA-β-gal assay measures the activity of β-gal expressed by senescent cells *in vitro* and *in vivo* that can be detected at pH. 6.0 by immune-histochemistry [12, 111]. It mainly depends increasing the lysosomal content of senescent cells [112] and not related to any of the major senescence pathways.

Not only is the detection of SA-β-gal positive cells *in vivo* associated with replicative senescence and a reduction in telomeric structural integrity, but also with premature senescence induced by genomic DNA damage and oncogenic stress p16 and ARF as markers of *in vivo* senescence [113].

### Senescence-associated heterochromatic foci (SAHF)

Senescent cells display heterochromatin condensation structures involved in the formation of heterochromatic foci [60]. These foci can be viewed on a microscope as condensed regions of DNA/chromatin, which appear as DAPI clusters. These SAHF suppress E2F transcription factor-regulated genes such as MCM3, PCNA or Cyclin A [114, 115]. Several proteins that accumulate at SAHF are valuable senescence markers such as HMGA, HIRA, HP1 and H3K9me3 in the in vitro senescence [115] and in vivo senescence [116].

### PML nuclear bodies

Promyelocytic leukemia protein (PML) is a tumor suppressor protein and its overexpression induces senescence [7,117,118], which involves cellular senescence in response to oncogenic stimuli such as Ras [7, 119, 120]. Moreover, PML levels are dependent upon DNA damage signals and it accumulates near unrepaired lesions of the genome [121]. PML bodies were frequently expressed in benign, but not malignant, prostate tumors [122].

### Lipofuscin granules

Lipofuscin is a highly oxidized cross-linked aggregate of protein and lipid clusters, and progressively accumulates in the cytoplasm of aged post-mitotic cells [123]. Further accumulation of it resists degradation by cellular proteolytic systems [124, 125]. The accumulation of lipofuscin granules in different organs including liver, lung, heart, muscle and nerve cells, is considered to be one of the wear and tear pigments and also, involves the pathway of macular degeneration, which is an aging-related malady [123].

Sudan black B stain (SBB) is used to identify lipofuscin as yellow granules. SBB is suitable for positively staining senescent cells and it is absent in SA-β-gal-negative cells [126]. SBB can be used with both frozen and paraffin-embedded tissues[36]; it is an easy assay to perform (no more than a few minutes), and it shares the common limitations with SA-β-gal staining.

Although significant effort has been made in the past decades to study senescence markers, none of these markers alone can serve as a true senescence indicator. As many of the identified markers were identified in specific conditions and whether they can be reliably used in different tissues and different models is still unknown (for additional discussion see the comprehensive, detailed review of senescence markers reported elsewhere [127]). It will be of great interesting if some of the lncRNAs and RBPs could be developed as new and insightful biomarkers for senescence.

## Modulation of senescence for therapeutic purpose and future directions

Most of the lncRNAs which are altered in senescence may play different roles including epigenetic alteration, modulating telomere length, histone modification, heterochromatin formation, alteration of proteostasis, and alteration of stem cells (see the comprehensive review demonstrating different lncRNAs disrupted in aging [128]). So, it seems that lncRNAs control the cellular homeostasis in different levels in cell development, differentiation, migration in the embryo till programmed cell death or cellular senescence.

Although the brain does not express replicative associated telomere shortening, at least at the same rate as other tissues, recent evidence indicates that telomeres could play a crucial role in brain biology [129]. Telomeres were shortened in patients with neurodegenerative diseases such as Alzheimer and other dementia diseases [130, 131]. Overexpression of telomerase in a telomerase-negative background provided rescue some of the pathologies that were attributed to telomere shortening and associated with the aging progression [129]. While postmitotic neurons are not capable of telomere elongation through replication because they do not divide, mitochondria-associated telomeres may be a novel target for telomerase to slow or repair telomere shortening in cells that do not divide or divide slowly [132-135].

Therefore, we sought to identify aging-associated lncRNAs (AALnc-RNAs) in mouse brain using young and old mice. Comparison of transcript expression patterns by RNA-Seq and microarray analysis, in young and old mice, revealed numerous AALnc-RNAs, including antisense transcripts, pseudogene-encoded transcripts, intergenic, sense-overlapping and other known lncRNAs (unpublished data).

Because cellular senescence is important in many age-associated processes, there is increased interest in understanding how to modulate senescence for therapeutic purposes. Traditional cancer chemotherapies have used compounds to elicit genotoxic insults to combat cancer by inducing extensive DNA damage [136], thereby killing the rapidly dividing tumor cells, but hopefully sparing the less rapidly dividing normal cells of the body. What is now apparent, and overlooked in the past, is how these standard therapies often trigger a potent senescence response cells as in the case of OIS. The contribution to senescence *in vitro* and *in vivo* of several cellular processes including activation of DDR, telomere shortening, SAHF formation, induction of ROS and autophagy also requires further detailed elucidation.

The delay of aging associated maladies and improvement of health may be afforded by the elimination of senescent cells, which is a goal of promising, therapy-driven investigations of senescence [137]. However, the observed functional improvement either related to the termination of further damage or damage reversal has yet to be realized. We believe that regulation of the effects of senescent cells, rather than their abundance, is more manageable and requires further investigation.

An alternative therapeutic approach was demonstrated by inhibition of the JAK protein, which mediates the action of some cytokines, represses SASP and alleviates frailty in old mice [138]. Rapamycin, a drug that is used as an immunosuppressant in humans, also robustly extends the lifespan of mice [139] and regulates the SASP [140, 141]. Also, assessment of telomere dynamics *in vivo* could be used as a meter for the aging progress, and therapies that help to maintain or restore telomeres are believed to have positive impacts on health and lifespan [129, 142]

Because identification of the factors secreted by senescent cells and contribute to aging is a priority, we imagine that lncRNA can be a target for senescent cells *in vivo* as has been the case *in vitro*. Also, it can be used for modulation of aging and aging-associated pathologies. With the rapid development of methods that daily uncover and characterize lncRNAs, one can imagine that lncRNAs can be used to control the senescent cell phenotype. Therefore, in light of increasing functional role of lncRNAs, the growing numbers of functional RNA elements should perhaps be considered. By utilizing new strategies, we hope to modulate disease pathways that have been previously considered to be intractable. Also, we need to uncover the role of lncRNAs in senescence during embryonic development and the difference in their expression profile compared to adults. The limited genomic annotation of lncRNAs and lack of robust protocols remains an obstacle.

In conclusion, the molecular mechanisms of senescence need more elucidation. Senescence forces cells into permanent withdrawal from the cell cycle and causes them to acquire distinct functional and morphologic changes that result in impaired cellular homeostasis. So, another challenge is the need to catalog in more detail the role of lncRNAs in senescence and whether they directly contribute to senescence. We also need to consider the following questions concerning the role of senescence during development and the possibility of lncRNAs as a link between senescence and apoptosis, as well as their exact role in adult stem cell proliferation, differentiation, and migration; at the same time, their potential involvement for therapeutic purposes.

When considering biology research today, cancer research ranked first, as cancer is the first cause of pathological death worldwide. On the other hand, we believe that aging research deserves the upper hand because it is inevitable all living creatures, if one does not die from aging, they will die from aging-associated diseases and cancer is one of them.

We hypothesize that aging is restricted to a finite period of time (i.e., lifespan) and aging is fated for all creatures. Are we able to stop the process of aging to keep our cells, tissues, and organs from losing function? If we succeed to delay or even cure aging-associated diseases, are we able to do the same with aging itself? Is aging a phenomenon that can be induced for certain therapeutic purposes and turned off if it is not needed? Indeed, aging is a mystery of life and requires more intensive research.
